# miR-21 Promotes Human Nucleus Pulposus Cell Proliferation through PTEN/AKT Signaling

**DOI:** 10.3390/ijms15034007

**Published:** 2014-03-05

**Authors:** Hongzhe Liu, Xiangwang Huang, Xiangyang Liu, Sheng Xiao, Yi Zhang, Tiecheng Xiang, Xiongjie Shen, Guoping Wang, Bin Sheng

**Affiliations:** Department of Orthopedic Surgery, Hunan Provincial People’s Hospital, Changsha 10005, China; E-Mails: guke11@yeah.net (H.L.); aiguke@yeah.net (X.L); kkllee1@yeah.net (S.X.); yyiiaa1@163.com (Y.Z.); bbjjaa@yeah.net (T.X.); aayyii1@126.com (X.S.); hahaii1@163.com (G.W.); kakaai@yeah.net (B.S.)

**Keywords:** disc degeneration, nucleus pulposus cells, miRNA, miR-21, proliferation

## Abstract

The precise role of nucleus pulposus cell proliferation in the pathogenesis of intervertebral disc degeneration remains to be elucidated. Recent findings have revealed that microRNAs, a class of small noncoding RNAs, may regulate cell proliferation in many pathological conditions. Here, we showed that miR-21 was significantly upregulated in degenerative nucleus pulposus tissues when compared with nucleus pulposus tissues that were isolated from patients with idiopathic scoliosis and that miR-10b levels were associated with disc degeneration grade. Moreover, bioinformatics target prediction identified PTEN as a putative target of miR-21. miR-21 inhibited PTEN expression by directly targeting the 3′UTR, and this inhibition was abolished through miR-21 binding site mutations. miR-21 overexpression stimulated cell proliferation and AKT signaling pathway activation, which led to cyclin D1 translation. Additionally, the increase in proliferation and cyclin D1 expression induced by miR-21 overexpression was almost completely blocked by Ly294002, an AKT inhibitor. Taken together, aberrant miR-21 upregulation in intervertebral disc degeneration could target PTEN, which would contribute to abnormal nucleus pulposus cell proliferation through derepressing the Akt pathway. Our study also underscores the potential of miR-21 and the PTEN/Akt pathway as novel therapeutic targets in intervertebral disc degeneration.

## 1. Introduction

Chronic low back pain affects >70% of people at some point in their lives, with approximately 10% becoming chronically disabled [1,2]. The causes of low back pain are multifactorial, and degenerative changes in the intervertebral disc (IVD) can contribute to the development of this pain [3]. The pathogenesis of intervertebral disc degenerative (IDD) disease has been ascribed to various etiological factors, including genetic predisposition, lifestyle, and aging [4]. However, the underlying cellular and molecular mechanisms of IDD need to be elucidated [5]. In this regard, there has been increasing evidence that supports a role for the formation of nucleus pulposus (NP) cell clusters and the proliferation of fibrocartilaginous tissue in IDD [6,7]. To date, the cause of increased NP cell proliferation in IDD remains unclear.

There is a growing body of evidence that supports the hypothesis that many cellular processes, including cell proliferation, apoptosis, and cytokine release, are regulated by a new class of 19–25 nucleotide, small, non-coding RNAs known as microRNAs (miRNAs) [8–10]. Gene expression of mRNAs is controlled by miRNAs through direct targeting, which leads to either translation repression or RNA degradation [11]. It has been estimated that miRNAs, which constitute only 1%–3% of the human genome, have the potential to regulate at least 20%–30% of all human genes [12]. miRNAs play crucial roles in diverse pathological conditions, including cancer, neurodegeneration, and aging [11,13,14]. However, the precise role of miRNAs in the pathogenesis of IDD remains to be elucidated.

miR-21 is one of the best studied miRNAs, and it is involved in the regulation of cell proliferation [15,16]. As a multi-functional miRNA, miR-21 is expressed in diverse tissue types [17–19]. The dysregulated expression of miR-21 is associated with malignant diseases, which are characterized by uncontrolled cell proliferation [20,21]. miR-21 has also been found to be associated with chondrocyte apoptosis, proliferation and cartilage matrix production [22]. Given that miR-21 is a crucial regulator of proliferation pathways and disorders characterized by abnormal proliferation, we hypothesized that miR-21 might play a role in the process of IDD. To date, only two studies have attempted to address the pathogenesis of IDD in relation to miRNAs [23,24]. Accordingly, the aim of this study was to investigate the role of miR-21 in IDD and to elucidate its molecular mechanism.

## 2. Results and Discussion

### 2.1. miR-21 Expression Is Up-Regulated in Degenerative NP Tissues and Correlates with Degeneration Grade

**Figure 1.**
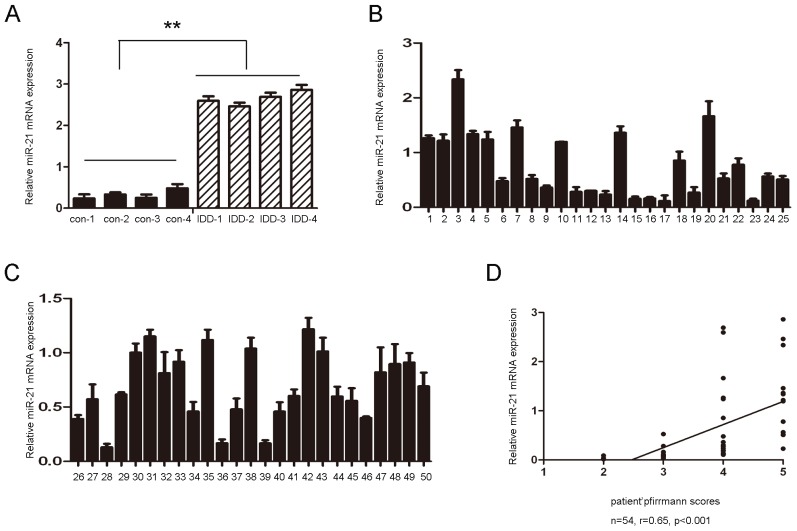
The expression of miR-21 in human nucleus pulposus tissues. (**A**) The expression of miR-21 in four degenerative nucleus pulposus tissues and four idiopathic scoliosis nucleus pulposus tissues. These degenerative NP tissues exhibited significantly higher expression of miR-21 compared to the control; (**B** and **C**) TaqMan RT-PCR analysis of miR-21 in nucleus pulposus tissue from other 50 patients; (**D**) The correlation between the expression of miR-21 and the disc degeneration grade of the patients. The error bars represent SD. ** indicates *p* < 0.01.

First, we examined whether miR-21 is differentially expressed in human degenerative NP tissues. The expression level of miR-21 in four human degenerative NP and normal NP tissues was determined using TaqMan real-time PCR. Compared with control tissues, our results demonstrated that the expression level of miR-21 was significantly upregulated in degenerative NP tissues (Figure 1A). To study the relationship between miR-21 and degenerative NP occurrence, the expression of miR-21 was measured in 50 clinical patients using Taqman real-time PCR. As shown in Table S1 and Figure 1, there were no significant differences observed between the samples from different herniation types or genders. The expression of miR-21 was positively correlated with the disc degeneration grade (*r* = 0.64, *p* < 0.001) but not with the duration of the symptoms or with the age of the patients.

### 2.2. miR-21-Induced NP Cell Proliferation and Cyclin D1 Expression

**Figure 2.**
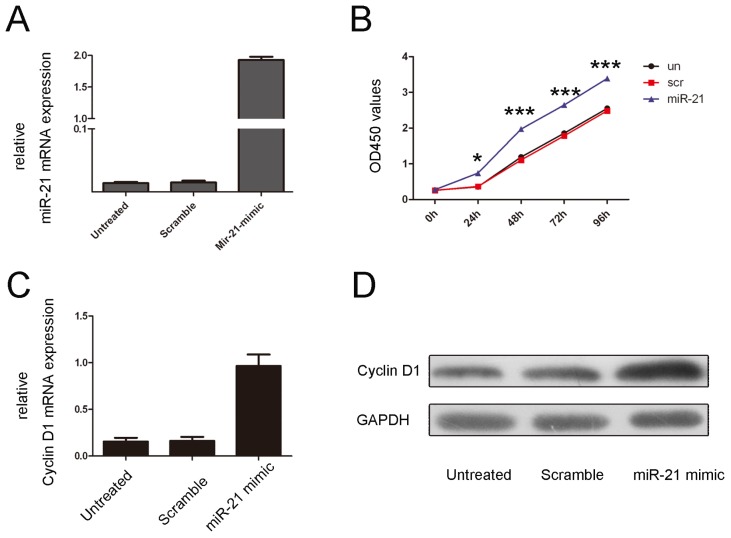
Overexpression of miR-21 promotes NP cell proliferation. (**A**) Expression levels of miR-21 were examined using real-time PCR for non-transfected cells or after transfection of 50 nmol/L of a miR-21 mimic or a scramble control; (**B**) The growth of NP cells is shown for non-transfected cells or after transfection with 50 nmol/L of a miR-21 mimic or a scramble control. The growth index was assessed 1, 2, 3 and 4 days post-transfection; (**C**) miR-21 promotes cyclin D1 mRNA expression. NP cells were transfected with 50 nmol/L of a miR-21 mimic or a scramble control or remained non-transfected. Cyclin D1 was detected using real-time PCR; (**D**) miR-21 promotes cyclin D1 protein expression. NP cells were transfected with 50 nmol/L of a miR-21 mimic or a scramble control or remained non-transfected. Cyclin D1 was detected by western blot analysis. GAPDH was detected as a loading control. The values are presented as the mean ± SD. Compared with the control, * *p* < 0.05 and *** *p* < 0.001.

Because the expression of miR-21 was associated with the disc degeneration grade of the patients, we examined the effects of miR-21 expression on NP cell proliferation. The NP cells were transfected with a scrambled control oligo or with a miR-21 mimic; all of the oligos had a high transfection efficiency (Figure 2A). A CCK-8 proliferation assay demonstrated that cell proliferation was increased in NO cells that were transfected with the miR-21 mimic compared with the scrambled oligo-transfected cells or untreated cells (Figure 2B). The proliferative effect of miR-21 was further confirmed by measuring cyclin D1 expression. As shown in Figure 2C,D, there was a significant increase in the protein and mRNA levels of cyclin D1 in miR-21 mimic-transfected group compared with the control group or the untreated group.

### 2.3. miR-21 Translationally Repressed PTEN

**Figure 3.**
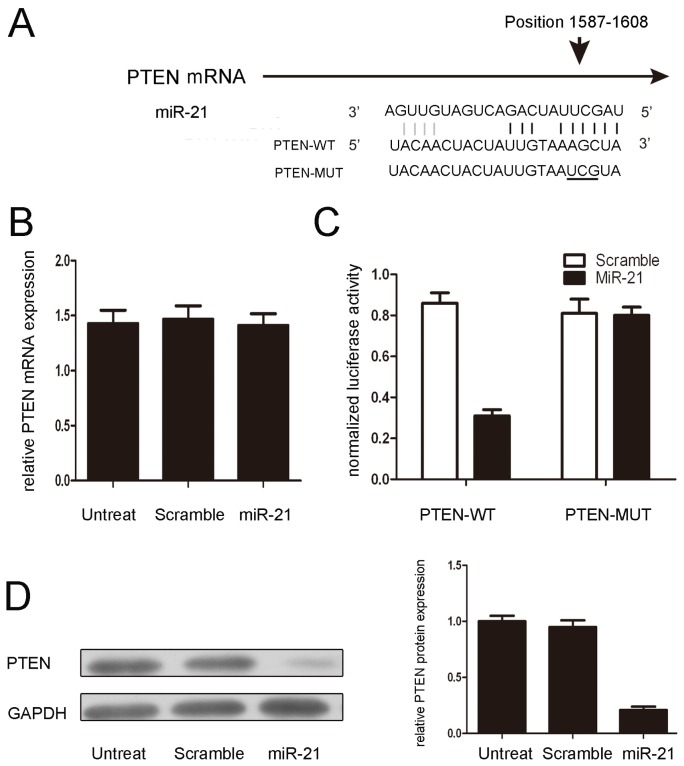
PTEN is a direct target of miR-21. (**A**) Predicted duplex formation between the human PTEN 3′UTR and miR-21. In the upper panel, the sequence alignment of miR-21 with the binding site of the PTEN 3′UTR is shown. In the lower panel, the sequence of the miR-10b binding site within the PTEN 3′UTR of three species is shown; (**B**) miR-21 does not alter the mRNA level of PTEN, as measured using real-time PCR; (**C**) The luciferase activity of wild-type (WT-UTR) or mutant (MUT-UTR) PTEN is shown; (**D**) PTEN protein expression in NP cells that were transfected with 50 nmol/L of a miR-21 mimic or a scramble control or remained non-transfected. The values are presented as the mean ± SD.

miRNAs influence biological functions by negatively regulating their target genes. It has been reported that PTEN is a direct target of miR-21 in breast carcinoma cells. As predicted by PicTar, there was complementarity between miR-21 and the PTEN 3′UTR (Figure 3A). miR-21 overexpression reduced the amount of protein but not the PTEN mRNA levels in NP cells (Figure 3B,D). Next, the effect of miR-21 on the translation of PTEN mRNA into protein was assessed using a luciferase reporter assay in NP cells (Figure 3C). miR-21 overexpression significantly reduced the luciferase activity of the reporter gene in the wild-type, but not mutant, PTEN 3′UTR, indicating that miR-21 directly targeted the PTEN 3′UTR.

### 2.4. miR-21 Induced Cell Proliferation through the Activation of PTEN/AKT Signaling

**Figure 4.**
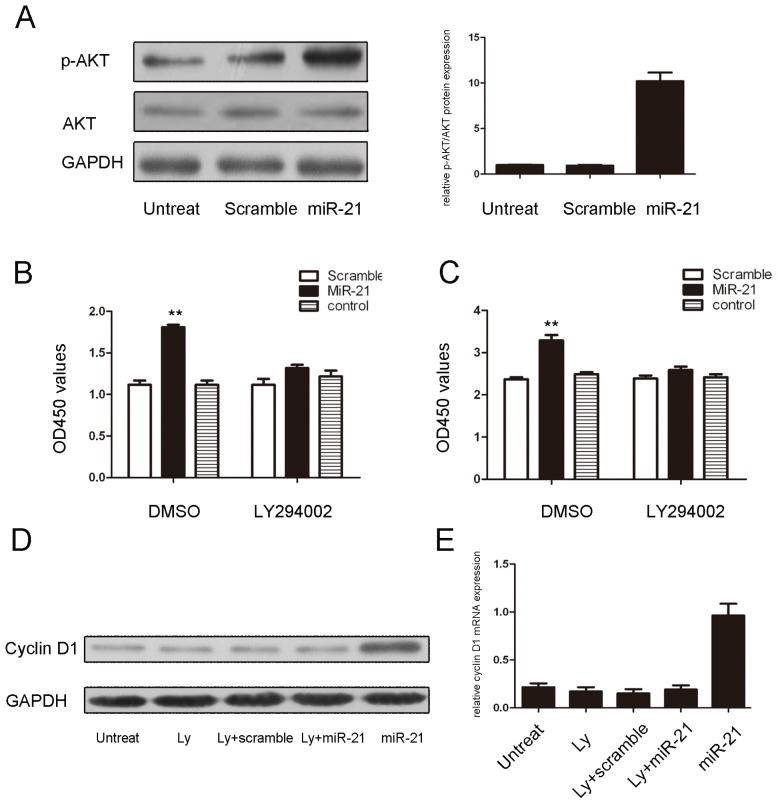
miR-21 induces cell proliferation through an AKT-dependent pathway. (**A**) miR-21 promotes Akt phosphorylation. NP cells were transfected with 50 nmol/L of a miR-21 mimic or a scramble control or remained non-transfected. AKT and p-AKT were detected by immunoblotting; (**B**) The proliferative effects of miR-21 overexpression were largely blocked by Ly294002, an AKT inhibitor, in NP cells that were transfected for 24 h with 50 nmol/L of a miR-21 mimic or a scramble control or remained non-transfected; (**C**) The proliferative effects of miR-21overexpression were largely blocked by Ly294002, an AKT inhibitor, in NP cells that were transfected for 96 h with 50 nmol/L of a miR-21 mimic or a scramble control or remained non-transfected. The values are presented as the mean ± SD; (**D**) Inhibition of AKT represses the miR-21-induced expression of cyclin D1 protein; (**E**) The inhibition of AKT represses the miR-21-induced expression of cyclin D1 mRNA. The values are presented as the mean ± SD. Compared with the control, ** *p* < 0.01.

The loss of PTEN expression or activity contributes to increased AKT activation and leads to subsequent growth and survival in many cell types. As shown in Figure 4A,B, miR-21 overexpression led to AKT phosphorylation in NP cells. Additionally, the proliferative role of miR-21overexpression and its effects on the expression of cyclin D1 were largely blocked by, an AKT inhibitor, Ly294002 (Figure 4C–E).

### 2.5. Discussion

miRNAs have been demonstrated to play an important role in diverse biological and pathological processes [25], including cell growth, differentiation, apoptosis and carcinogenesis. However, the potential roles of miRNAs in disc degeneration remain largely uncharacterized. In the current study, miR-21 was found to be upregulated in human degenerative NP tissues when compared with normal NP tissues. Moreover, the overexpression of miR-21 increased NP cell proliferation. Mechanistically, the overexpression of miR-21 led to increased p-Akt signaling by directly targeting PTEN. Furthermore, the proliferative effect and the increased expression of cyclin D1 expression after miR-21 overexpression in human NP cells was almost completely blocked by Ly294002, an AKT inhibitor. These findings suggest that miR-21 and the PTEN/Akt pathway may potentially be novel therapeutic targets in the treatment of IDD.

miR-21, a well-known miRNA, is most frequently overexpressed in several different types of human cancers, and it has been shown to be implicated in multiple cell processes, including cell proliferation, apoptosis, and invasion [26,27]. The dramatic upregulation of miR-21 has also been reported in most tumor types, including neuroblastoma, lung, breast, colorectal, pancreas, and lymphoma [27–32]. However, the level of miR-21 in degenerative NP tissues and its role in the pathogenesis of IDD are unknown. In our study, miR-21 levels were significantly increased in tissues from patients with degenerative NP, and miR-21 was significantly associated with disc degeneration grade (Figure 1). The elevated levels of miR-21 may be due to local inflammation and associated post-trauma reactions in the intervertebral disc. Previous studies have shown that miR-21 was upregulated in response to PDGF treatment. The precise mechanism of miR-21 overexpression still needs to be elucidated. To further study the function of miR-21 in the development of IDD, miR-21 was overexpressed by transfecting a miR-21 mimic into NP cells. miR-21 overexpression significantly increased NP cell proliferation (Figure 2). Previous studies have shown that NP cell clusters and the proliferation of fibrocartilaginous tissue contribute to the development of IDD. These findings suggest that increased NP cell proliferation induced by the upregulation of miR-21 may be a possible mechanism in IDD development.

Previous studies indicated that miR-21 repressed PTEN through translational inhibition and that the miR-21 binding site in the PTEN 3′UTR is crucial [21,33]. In agreement with these findings, we found that miR-21 interfered with the translation of PTEN without reducing its mRNA level, and the vector expressing a mutated PTEN 3′UTR was resistant to miR-21 inhibition in NP cells (Figure 3). Restored expression of PTEN also abrogated the induction of NP cell proliferation that was caused by miR-21 overexpression. Moreover, the regulation of PTEN by miR-21 in NP cells was further corroborated by the observation that miR-21 overexpression reduced PTEN protein expression. These findings indicated that miR-21 promoted the proliferation of NP cells by directly targeting PTEN.

PTEN is a key molecule in the development of many diseases because PTEN modulates cell proliferation, survival, apoptosis and metabolism via its target molecules, phosphoinositide-3kinase (PI3K) and Akt [34]. Previous studies have shown that the loss of PTEN expression or activity contributes to increased Akt activation and subsequent growth and survival in many cell types [35]. Our data presented in the current study indicate that the overexpression of miR-21 leads to the activation of AKT in NP cells. Moreover, the proliferative effects of miR-21 overexpression were largely blocked by an AKT inhibitor, Ly294002 (Figure 4). Taken together, our results suggest that the PTEN/AKT signaling pathway is an important target of miR-21in NP cells.

## 3. Experimental Section

### 3.1. Ethics Statement

All of the experimental protocols were approved by the Clinical Research Ethics Committee of the Hunan Provincial People’s Hospital. Human lumbar IVD samples were obtained from patients undergoing discectomy following approval from the Clinical Research Ethics Committee of the Hunan Provincial People’s Hospital, with fully informed, written consent from the patients.

### 3.2. Patients and SAMPLES

Human lumbar NP specimens were collected from patients with idiopathic scoliosis (*n* = 4; average age 20 ± 1.53, range 18–22 years) and from patients with IDD (*n* = 54; average age 46.18 ± 7.84, range 29–56 years). Routine MRI scans of the lumbar spine were taken of these patients before the operation; the degree of disc degeneration was graded from the T2-weighted images using a modified Pfirrmann classification [36].

### 3.3. Isolation and Primary Culture of Human NP Cells

NP cells were isolated as previously described [7,37]. The tissue specimens were first washed twice with PBS, and the NP was separated from the AF using a stereotaxic microscope, cut into pieces (2–3 mm^3^), and the NP cells were released from the NP tissues by incubation with 0.25 mg/mL type II collagenase (Invitrogen, Carlsbad, CA, USA) for 12 h at 37 °C in Dulbecco’s modified Eagle medium (DMEM; GIBCO, Grand Island, NY, USA). After isolation, the NP cells were resuspended in DMEM containing 10% FBS (GIBCO), 100 μg/mL streptomycin, 100 U/mL penicillin and 1% l-glutamine and then incubated at 37 °C in a humidified atmosphere with 95% (*v*/*v*) air and 5% (*v*/*v*) CO_2_. The confluent cells were detached by trypsinization, seeded into 35 mm tissue culture dishes in complete culture medium (DMEM supplemented with 10% FBS, 100 μg/mL streptomycin and 100 U/mL penicillin), and incubated in a 37 °C, 5% CO_2_ (*v*/*v*) environment. The medium was changed every two days. The second passage was used for subsequent experiments.

### 3.4. Dual Luciferase Assays

The NP cells were co-transfected with 0.4 μg of the reporter construct, 0.2 μg of the pGL-3 control vector, and miR-21 or negative controls. The cells were harvested 24 h post-transfection and assayed using a Dual Luciferase Assay (Promega, Wisconsin-Madison, WI, USA) according to the manufacturer’s instructions. All of the transfection assays were carried out in triplicate.

### 3.5. Oligonucleotides, Constructs, and Transfections

The mimics of miR-21 and the negative controls were purchased from Dharmacon (Chicago, IL, USA) and transfected into NP cells using DharmaFECT 2 (Chicago, IL, USA) at a final concentration of 100 nM.

### 3.6. RNA Isolation, Reverse Transcription and Quantitative RT-PCR

Total RNA was extracted from the harvested cells and tissues using Trizol reagent (Invitrogen, Carlsbad, CA, USA) according to the manufacturer’s instruction. Complementary DNAs (cDNAs) of the mRNAs were obtained using M-MLV reverse transcriptase (Invitrogen, Carlsbad, CA, USA) and oligo(dT)18. For microRNAs, the stem-loop reverse transcription was manufactured according to Chen’s report. The primers for reverse transcription of the microRNAs, quantitative RT-PCR, and the Taqman probes are described in Table S2. Quantitative RT-PCR was conducted in a Bio-Rad (Hercules, CA, USA) IQ5 Real-Time PCR system according to the manufacturer’s instruction. The data were normalized using endogenous GAPDH and U6 snRNA for mRNA and miRNAs, respectively. The 2^−ΔΔCt^ method was used for the analysis of the PCR data.

### 3.7. Western Blotting

Western blot analysis was conducted using standard methods. Proteins were separated on a 10% SDS-PAGE gel and then transferred to PVDF membranes (Amersham, Buckinghamshire, UK). The membranes were blocked overnight using 5% non-fat dried milk and incubated for 2 h with an anti-PTEN antibody (Abcam, Cambridge, UK; 1:1000), anti-AKT antibody (Bioworlde, Minneapolis, MN, USA; 1:1000), anti-p-AKT antibody (Bioworlde, Minneapolis, MN, USA; 1:1000), or anti-GAPDH antibody (Proteintech, Chicago, IL, USA; 1:50,000). After washing with TBST (10 mM Tris, pH 8.0, 150 mM NaCl, and 0.1% Tween20), the membranes were incubated for 2 h with a goat anti-rabbit antibody (ZSGB-BIO, Beijing, China; 1:5000 or 1:50,000).

### 3.8. Cell Proliferation

Cells were re-seeded into 96-well plates at a density of 10,000 cells per well after transfection or infection for 12 h. Cell viability was measured every 24 h by adding 10% CCK-8 (DOJINDO, Tokyo, Japan) and incubating at 37 °C for 3 h. The optical density was read at 450 nm using a microplate spectrophotometer. Each experiment was performed in triplicate.

### 3.9. Statistical Analysis

Statistical analyses were performed using the SPSS 17.0 statistical software program (17.0; IBM, Chicago, IL, USA). For the human studies, the Kruskal-Wallis test was used to assess the difference in expression of miR-21 in disc specimens from different herniation types, and an independent *t*-test was used to assess the difference between specimens from different genders. The correlation between the expression of miR-21 and the age and BMI of the patients was determined by Pearson’s test. The difference between the expression of miR-21 and the duration of the symptoms was determined by Spearman’s test. The data were expressed as the mean ± SD. Western blot results were normalized using GAPDH. Independent experiments were performed twice. Statistical analysis was performed using Student’s *t*-test. *p* values less than 0.05 were considered statistically significant.

## 4. Conclusions

Our data suggests that miR-21 is overexpressed in human degenerative NP tissues and that its level is positively associated with disc degeneration grade. In addition, miR-21 overexpression increased NP cell proliferation by targeting PTEN, which derepressed Akt signaling. These results have shed new light on the role of miR-21 in the pathogenesis of IDD, and identified novel therapeutic targets for inhibiting abnormal NP cell proliferation in IDD.

## Supplementary Information



## References

[1] Frymoyer J.W., Cats-Baril W.L. (1991). An overview of the incidences and costs of low back pain. Orthop. Clin. N. Am..

[2] Nakamura M., Nishiwaki Y., Ushida T., Toyama Y. (2011). Prevalence and characteristics of chronic musculoskeletal pain in Japan. J. Orthop. Sci..

[3] Samartzis D., Karppinen J., Mok F., Fong D.Y., Luk K.D., Cheung K.M. (2011). A population-based study of juvenile disc degeneration and its association with overweight and obesity low back pain and diminished functional status. J. Bone Jt. Surg. Am..

[4] Hangai M., Kaneoka K., Kuno S., Hinotsu S., Sakane M., Mamizuka N., Sakai S., Ochiai N. (2008). Factors associated with lumbar intervertebral disc degeneration in the elderly. Spine J..

[5] Katz J.N. (2006). Lumbar disc disorders and low-back pain: Socioeconomic factors and consequences. J. Bone Jt. Surg. Am..

[6] Johnson W.E., Eisenstein S.M., Roberts S. (2001). Cell cluster formation in degenerate lumbar intervertebral discs is associated with increased disc cell proliferation. Connect. Tissue Res..

[7] Li Z., Shen J., Wu W.K., Yu X., Liang J., Qiu G., Liu J. (2012). Leptin induces cyclin D1 expression and proliferation of human nucleus pulposus cells via JAK/STAT PI3K/Akt and MEK/ERK pathways. PLoS One.

[8] Farazi T.A., Spitzer J.I., Morozov P., Tuschl T. (2011). miRNAs in human cancer. J. Pathol..

[9] Ferland-McCollough D., Ozanne S.E., Siddle K., Willis A.E., Bushell M. (2010). The involvement of microRNAs in Type 2 diabetes. Biochem. Soc. Trans..

[10] Mo Y.Y. (2012). MicroRNA regulatory networks and human disease. Cell. Mol. Life Sci..

[11] Yates L.A., Norbury C.J., Gilbert R.J. (2013). The long and short of microRNA. Cell.

[12] Chou J., Shahi P., Werb Z. (2013). MicroRNA-mediated regulation of the tumor microenvironment. Cell Cycle.

[13] Xie Z.R., Yang H.T., Liu W.C., Hwang M.J. (2007). The role of microRNA in the delayed negative feedback regulation of gene expression. Biochem. Biophys. Res. Commun..

[14] Roy S., Sen C.K. (2011). MiRNA in innate immune responses: Novel players in wound inflammation. Physiol. Genomics.

[15] Orso F., Balzac F., Marino M., Lembo A., Retta S.F., Taverna D. (2013). miR-21 coordinates tumor growth and modulates KRIT1 levels. Biochem. Biophys. Res. Commun..

[16] Zhang Y.X., Yue Z., Wang P.Y., Li Y.J., Xin J.X., Pang M., Zheng Q.Y., Xie S.Y. (2013). Cisplatin upregulates MSH2 expression by reducing miR-21 to inhibit A549 cell growth. Biomed. Pharmacother..

[17] Toiyama Y., Takahashi M., Hur K., Nagasaka T., Tanaka K., Inoue Y., Kusunoki M., Boland C.R., Goel A. (2013). Serum miR-21 as a diagnostic and prognostic biomarker in colorectal cancer. J. Natl. Cancer Inst..

[18] Vicinus B., Rubie C., Stegmaier N., Frick V.O., Kolsch K., Kauffels A., Ghadjar P., Wagner M., Glanemann M. (2013). miR-21 and its target gene CCL20 are both highly overexpressed in the microenvironment of colorectal tumors: Significance of their regulation. Oncol. Rep..

[19] Wang P., Zhuang L., Zhang J., Fan J., Luo J., Chen H., Wang K., Liu L., Chen Z., Meng Z. (2013). The serum miR-21 level serves as a predictor for the chemosensitivity of advanced pancreatic cancer and miR-21 expression confers chemoresistance by targeting FasL. Mol. Oncol..

[20] Wang N., Zhang C.Q., He J.H., Duan X.F., Wang Y.Y., Ji X., Zang W.Q., Li M., Ma Y.Y., Wang T. (2013). MiR-21 down-regulation suppresses cell growth invasion and induces cell apoptosis by targeting FASL TIMP3 and RECK genes in esophageal carcinoma. Dig. Dis. Sci..

[21] Xiong B., Cheng Y., Ma L., Zhang C. (2013). MiR-21 regulates biological behavior through the PTEN/PI-3 K/Akt signaling pathway in human colorectal cancer cells. Int. J. Oncol..

[22] Kongcharoensombat W., Nakasa T., Ishikawa M., Nakamae A., Deie M., Adachi N., Mohamed A., Ochi M. (2010). The effect of microRNA-21 on proliferation and matrix synthesis of chondrocytes embedded in atelocollagen gel. Knee Surg. Sports Traumatol. Arthrosc..

[23] Wang H.Q., Yu X.D., Liu Z.H., Cheng X., Samartzis D., Jia L.T., Wu S.X., Huang J., Chen J., Luo Z.J. (2011). Deregulated miR-155 promotes Fas-mediated apoptosis in human intervertebral disc degeneration by targeting FADD and caspase-3. J. Pathol..

[24] Yu X., Li Z., Shen J., Wu W.K., Liang J., Weng X., Qiu G. (2013). MicroRNA-10b promotes nucleus pulposus cell proliferation through RhoC-Akt pathway by targeting HOXD10 in intervetebral disc degeneration. PLoS One.

[25] Van Wynsberghe P.M., Chan S.P., Slack F.J., Pasquinelli A.E. (2011). Analysis of microRNA expression and function. Methods Cell Biol..

[26] Bhagat T.D., Zhou L., Sokol L., Kessel R., Caceres G., Gundabolu K., Tamari R., Gordon S., Mantzaris I., Jodlowski T. (2013). miR-21 mediates hematopoietic suppression in MDS by activating TGF-beta signaling. Blood.

[27] Capodanno A., Boldrini L., Proietti A., Ali G., Pelliccioni S., Niccoli C., D’Incecco A., Cappuzzo F., Chella A., Lucchi M. (2013). Let-7g and miR-21 expression in non-small cell lung cancer: Correlation with clinicopathological and molecular features. Int. J. Oncol..

[28] Schee K., Boye K., Abrahamsen T.W., Fodstad O., Flatmark K. (2012). Clinical relevance of microRNA miR-21 miR-31 miR-92a miR-101 miR-106a and miR-145 in colorectal cancer. BMC Cancer.

[29] Seca H., Lima R.T., Lopes-Rodrigues V., Guimaraes J.E., Almeida G.M., Vasconcelos M.H. (2013). Targeting miR-21 induces autophagy and chemosensitivity of leukemia cells. Curr. Drug Targets.

[30] Sicard F., Gayral M., Lulka H., Buscail L., Cordelier P. (2013). Targeting miR-21 for the therapy of pancreatic cancer. Mol. Ther..

[31] Tao T., Wang Y., Luo H., Yao L., Wang L., Wang J., Yan W., Zhang J., Wang H., Shi Y. (2013). Involvement of FOS-mediated miR-181b/miR-21 signalling in the progression of malignant gliomas. Eur. J. Cancer.

[32] Soria-Valles C., Gutierrez-Fernandez A., Guiu M., Mari B., Fueyo A., Gomis R.R., Lopez-Otin C. (2013). The anti-metastatic activity of collagenase-2 in breast cancer cells is mediated by a signaling pathway involving decorin and miR-21. Oncogene.

[33] Yang S.M., Huang C., Li X.F., Yu M.Z., He Y., Li J. (2013). miR-21 confers cisplatin resistance in gastric cancer cells by regulating PTEN. Toxicology.

[34] Chappell W.H., Steelman L.S., Long J.M., Kempf R.C., Abrams S.L., Franklin R.A., Basecke J., Stivala F., Donia M., Fagone P. (2011). Ras/Raf/MEK/ERK and PI3K/PTEN/Akt/mTOR inhibitors: rationale and importance to inhibiting these pathways in human health. Oncotarget.

[35] Hyun T., Yam A., Pece S., Xie X., Zhang J., Miki T., Gutkind J.S., Li W. (2000). Loss of PTEN expression leading to high Akt activation in human multiple myelomas. Blood.

[36] Pfirrmann C.W., Metzdorf A., Zanetti M., Hodler J., Boos N. (2001). Magnetic resonance classification of lumbar intervertebral disc degeneration. Spine.

[37] Li Z., Shen J., Wu W.K., Yu X., Liang J., Qiu G., Liu J. (2013). The role of leptin on the organization and expression of cytoskeleton elements in nucleus pulposus cells. J. Orthop. Res..

