# Supplementary material for: miR-21 Promotes Human Nucleus Pulposus Cell Proliferation through PTEN/AKT Signaling
Source: Int J Mol Sci. 2014 Mar 5;15(3):4007–18. doi: 10.3390/ijms15034007 (PMC3975380; doi:10.3390/ijms15034007)
Supplement: Supplementary file 1 [file ijms-15-04007-s001.pdf]

Supplementary Information

Table S1. Clinical Finding in 54 Patients with LDH.

Patient No.	Sex/Age (year)	Type of LDH	Level	Duration of Symptoms(mo)	MRI Scores
1	F/53	SE	L5/S1	9	4.
2	M/54	P	L4/L5	15	5.
3	M/56	S	L3/L4	25	5.
4	F/42	TE	L4/L5	24	5.
5	M/39	S	L5/S1	16	4.
6	F/44	P	L4/L5	34	4.
7	M/56	P	L4/L5	12	5.
8	M/42	SE	L5/S1	12	5.
9	F/39	TE	L5/S1	12	4.
10	M/46	SE	L4/L5	21	5.
11	F/53	TE	L4/L5	5	3.
12	M/36	TE	L5/S1	1	4.
13	F/46	P	L4/L5	28	4.
14	M/51	S	L5/S1	26	5.
15	F/41	P	L5/S1	24	3.
16	F/42	SE	L4/L5	36	3.
17	M/33	TE	L5/S1	21	3.
18	F/55	SE	L3/L4	24	4.
19	M/51	P	L4/L5	21	4.
20	M/56	S	L3/L4	31	4.
21	F/46	S	L4/L5	34	3.
22	M/56	SE	L5/S1	18	5.
23	F/42	P	L4/L5	13	4.
24	M/34	S	L5/S1	17	5.
25	M/55	P	L4/L5	20	5.
26	M/44	S	L3/L4	12	2.
27	M/46	SE	L5/S1	36	4.
28	M/31	P	L3/L4	16	2.
29	M/32	SE	L4/L5	3	2.
30	F/36	TE	L3/L4	13	3.
31	M/46	P	L3/L4	7	3.
32	F/57	P	L5/S1	18	4.
33	M/54	SE	L5/S1	15	4.
34	F/43	P	L4/L5	9	3.
35	M/42	TE	L5/S1	11	3.
36	F/38	S	L3/L4	3	3.
37	F/45	S	L4/L5	18	3.
38	M/52	S	L5/S1	6	3.

Table S1. *Cont.*

Patient No.	Sex/Age (year)	Type of LDH	Level	Duration of Symptoms(mo)	MRI Scores
39	F/29	P	L4/L5	6	2.
40	F/48	SE	L4/L5	11	3.
41	F/54	TE	L5/S1	6	3.
42	M/48	SE	L3/L4	19	4.
43	F/55	TE	L4/L5	18	4.
44	M/38	TE	L4/L5	8	2.
45	F/54	SE	L5/S1	12	4.
46	F/36	S	L3/L4	12	2.
47	M/47	TE	L5/S1	25	5.
48	M/48	P	L4/L5	31	4.
49	M/41	TE	L5/S1	29	3.
50	M/56	P	L3/L4	23	4.
51	F/55	TE	L3/L4	15	4.
52	M/47	P	L4/L5	21	5.
53	M/51	S	L5/S1	23	4.
54	M/53	TE	L4/L5	20	5.

F, indicates female; LDH, lumbar disc herniation; M, male; P, protrusion; S, sequestration; SE, subligamentous extrusion; TE, transligamentous extrusion; L, lumbar; S, sacral; MRI, magnetic resonance imaging.

Table S2. Primer/mimics/probe sequence.

Name	Sequence (5'–3')
miRNA reverse transcription prime	
miRNA-21	CGTCGCTACATCGAGTGTAGCATATGCGACGTCAACATC
U6 snRNA	AAAATATGGAACGCTTCACGAATTTG
Real-time PCR primer sequence	
miRNA-21	TGTCGGGTAGCTTATCAGAC
	TTCAGACAGCCCATCGACTG
U6 snRNA	CTCGCTTCGGCAGCACATATACT
	ACGCTTCACGAATTTGCGTGTC
PTEN	GCGTGCAGATAATGACAAGG
	GGATTTGACGGCTCCTCTAC
GAPDH	CAAGGTCATCCATGACAACTTTG
	GTCCACCACCCTGTTGCTGTAG
miRNA Mimics sequence	
miRNA-21	UAGCUUAUCAGACUGAUGUUGA
	AACAUCAGUCUGAUAAGCUAUU
Negtive control	UUCUCCGAACGUGUCACGUTT
	ACGUGACACGUUCGGAGAATT
